# Electrophysiology of the Camel Heart: A Systematic Review of Evidence from *Camelus dromedarius*

**DOI:** 10.3390/biology15191711

**Published:** 2026-09-26

**Authors:** Alyazia Alshamsi, Jawaher Abdulhakim, Mariam Alnuaimi, Azhar T. Rahma, Reem AlSheryani, Ismail Elkonaisi, Frank C. Howarth

**Affiliations:** 1 Department of Physiology, College of Medicine & Health Sciences, United Arab Emirates University, Al Ain P.O. Box 15551, United Arab Emirates; 202311682@uaeu.ac.ae (A.A.); 202315522@uaeu.ac.ae (J.A.); 202308559@uaeu.ac.ae (M.A.); 2 Institute of Public Health, College of Medicine & Health Sciences, United Arab Emirates University, Al Ain P.O. Box 15551, United Arab Emirates; azhar.talal@uaeu.ac.ae (A.T.R.); i.konaisi@uaeu.ac.ae (I.E.); 3 National Medical Library, College of Medicine & Health Sciences, United Arab Emirates University, Al Ain P.O. Box 15551, United Arab Emirates; reem.alsheryani@uaeu.ac.ae

**Keywords:** dromedary camel, electrocardiography, atrioventricular conduction, arrhythmia, anaesthesia, telemetry, systematic review

## Abstract

**Objective:** We aimed to synthesise the available evidence on dromedary-camel electrocardiographic (ECG) measurements, rhythm, and conduction while distinguishing direct electrophysiological studies from supporting cardiovascular reports. **Methods:** PubMed/MEDLINE, Scopus, Embase, Web of Science, Google Scholar, and supplementary sources were searched from inception to 22 December 2025. During revision, the research librarian verified that the saved strategies contained the principal ECG and conduction terms. This verification did not retrieve or screen additional database records. **Results:** Fifteen primary studies met the eligibility criteria, all involving *Camelus dromedarius*. Six provided direct ECG or electrical telemetry data, whereas nine provided supporting heart rate, Doppler, echocardiographic, or haemodynamic data that were not used to derive ECG intervals or arrhythmia frequency. Direct studies reported low resting heart rates, atrioventricular conduction times (PR intervals) of approximately 180–260 ms, and drug-associated rhythm or conduction abnormalities. Lead configuration, animal position, recording duration, sampling, and outcome reporting varied substantially. Biological sex was incompletely reported, and no study compared males with females. **Conclusions:** The directly measured ECG findings are preliminary descriptors rather than validated reference intervals. Confidence is limited by small samples, methodological heterogeneity, the dated search, and possible loss of baseline studies through the executed exposure/context block. The conclusions apply only to *Camelus dromedarius*. Registration: International Prospective Register of Systematic Reviews (PROSPERO), CRD420251169514.

## Simple Summary

Electrocardiography (ECG) is used to assess cardiac rate, rhythm, conduction, and repolarisation, but evidence in camels remains limited. This review identified 15 primary studies in dromedary camels: six reported direct ECG or electrical telemetry data and nine reported supporting heart rate or haemodynamic outcomes. Direct studies repeatedly described low resting heart rates, PR intervals of approximately 180–260 ms, and drug-associated rhythm or conduction abnormalities. The evidence base was limited, and most studies enrolled small samples and used inconsistent lead configurations, animal positions, recording durations, and reporting methods; biological sex was also often unreported. Future studies should use standardised digital ECG protocols, longer rhythm monitoring, and adequately powered, sex-diverse cohorts. No eligible Bactrian-camel study was identified, and the summarised values should not be treated as validated reference limits.

## 1. Introduction

The dromedary camel (*Camelus dromedarius*) has a four-chambered mammalian heart with sinoatrial, atrioventricular, His-bundle, bundle-branch, and Purkinje components that generate and distribute cardiac electrical activity. Electrocardiography (ECG) records the resulting P wave, PR interval, QRS complex, ST segment, T wave, and RR interval, providing non-invasive information on rate, rhythm, conduction, and repolarisation. Foundational single-animal reports described low heart rates, long atrioventricular conduction times, variable QRS morphology and axis, and discordant T-wave patterns [1,2].

The largest baseline cohort used a base–apex lead and demonstrated age-related variation in heart rate and conduction intervals [3]. Across the literature, measured values may also be influenced by lead configuration, electrode placement, animal position, restraint, recording duration, equipment, filtering, and beat selection. In this review, telemetry was considered direct evidence only when it recorded cardiac electrical activity during movement or exercise rather than heart rate alone [4].

Pharmacological studies indicate that dromedary cardiac rate and conduction are responsive to autonomic modulation. Xylazine and detomidine were associated with bradycardia, sinus arrhythmia, and sinoatrial or atrioventricular conduction disturbances [5,6]. Chloral hydrate was associated with marked rate and conduction changes [7], whereas furosemide produced no statistically significant change in the reported ECG indices under the studied conditions [8].

Other studies reported heart rate, cardiac output, Doppler flow, or echocardiographic measurements without complete ECG intervals, waveforms, or rhythm assessment. Examples include propofol total intravenous anaesthesia, baseline echocardiography, and sedative Doppler studies [9,10,11]. These reports provide cardiovascular context but are not equivalent to direct electrophysiological evidence. A separate atrial-fibrillation case report was retained only as clinical context because case reports cannot estimate frequency and were excluded from the systematic dataset [12].

Biological sex was recorded when reported, but sex reporting was incomplete and no eligible study was designed or powered to compare males with females [3,6,8]. Consequently, this review does not infer sex-specific electrophysiological differences. Histological and developmental publications were also excluded from the core synthesis unless they contained original, extractable ECG or electrical telemetry outcomes.

The principal contribution of this review is the explicit separation of six studies with direct ECG or electrical telemetry outcomes from nine studies that provided only supporting heart rate or haemodynamic evidence. It also evaluates how acquisition methods and methodological quality affect confidence in the reported values, documents gaps in sex reporting and Bactrian-camel evidence, and avoids presenting heterogeneous numerical findings as clinical reference intervals.

This review aimed to synthesise ECG measurements, rhythm and conduction findings, pharmacological influences, and exercise responses in *Camelus dromedarius*; to distinguish direct electrophysiological evidence from contextual cardiovascular evidence; and to identify methodological priorities for future camel ECG research.

## 2. Materials and Methods

### 2.1. Protocol Registration and Reporting

This systematic review was prospectively registered with the International Prospective Register of Systematic Reviews (PROSPERO; CRD420251169514) before database searching and was reported in accordance with the Preferred Reporting Items for Systematic Reviews and Meta-Analyses (PRISMA) 2020 [13]. It synthesised previously published evidence and did not involve new animal recruitment, intervention, or access to identifiable data; therefore, new institutional animal-ethics approval was not required. During eligibility verification, one narrative review retained in error was removed under the prespecified exclusion of review articles. This correction applied the original study-design criterion and did not constitute a protocol amendment.

### 2.2. Eligibility Criteria

Eligibility was defined using the Population, Exposure, Comparator, and Outcome (PECO) framework. Biological sex, rather than gender, was recorded when it was reported by the source study.

#### 2.2.1. Population

The registered population comprised *Camelus dromedarius* and *Camelus bactrianus* of any age, biological sex, health status or geographic origin. No eligible *Camelus bactrianus* study was identified, so the synthesis and conclusions are restricted to *Camelus dromedarius*.

#### 2.2.2. Exposure and Comparator

Studies under resting conditions or a defined physiological, environmental, clinical, exercise, sedative, anaesthetic or pharmacological exposure were eligible. A comparator was not mandatory because many reports were descriptive.

#### 2.2.3. Outcomes

Core outcomes were heart rate and rhythm derived from ECG or electrical telemetry; the PR interval, measured from the onset of atrial depolarisation to the onset of ventricular depolarisation and used as an index of atrioventricular conduction; QRS duration, representing ventricular depolarisation; QT or corrected QT (QTc), representing ventricular depolarisation and repolarisation; waveform morphology; electrical axis; conduction abnormalities; arrhythmias; and heart rate variability. Telemetry was eligible as direct evidence only when it captured cardiac electrical signals. Studies reporting only clinical heart rate, cardiac output, Doppler, or echocardiographic outcomes were retained as contextual cardiovascular evidence and were not used to derive ECG values or arrhythmia frequency.

#### 2.2.4. Study Designs

Eligible experimental studies assigned an intervention or exposure, whereas observational studies recorded naturally occurring or clinical data without investigator assignment. Randomised trials, non-randomised experiments, crossover trials, cross-sectional studies, and retrospective clinical series were classified according to the design reported in each source. Single-animal reports, narrative or comparative reviews, systematic reviews, editorials, and conference abstracts without sufficient original data were excluded from the systematic synthesis; excluded publications could be cited only as clearly labelled context.

#### 2.2.5. Language and Time Frame

The searches covered database inception to 22 December 2025. English-language eligibility was applied at full-text screening, and database limits are reproduced exactly in Supplementary File S1. No search was conducted after 22 December 2025.

No protocol amendments were made. Removal of the ineligible review article applied the original study-design criterion rather than changing the protocol.

### 2.3. Information Sources

Electronic searches were conducted in PubMed/MEDLINE, Scopus, Web of Science Core Collection and Embase. Google Scholar, backward citation searching, manual journal searching and subject-expert input were used as supplementary sources.

The complete database-specific strategies actually used are reproduced in Supplementary File S1 together with the record counts imported into the screening dataset.

After the first review round, the research librarian rechecked the saved strategies and confirmed that PR interval, QT, QRS, heart rate variability, cardiac conduction, electrophysiology, and arrhythmia terms were present. This verification did not constitute an updated search: no additional database records were retrieved, deduplicated, or screened after 22 December 2025.

### 2.4. Search Strategy

The search strategy was developed with a research librarian and adapted to the controlled vocabulary, field codes, and syntax of each database. Boolean operators, phrase searching, and truncation were used as appropriate. Baseline or resting studies were eligible at screening, although the executed search also required an exposure/context block. The complete, unaltered database-specific strategies are reproduced in Supplementary File S1.

The complete strategies, platform limits and record counts used in the PRISMA flow are provided in Supplementary File S1. The final search date remained 22 December 2025.

Because the exposure/context block required terms related to dehydration, environmental stress, exercise, anaesthesia, or sedation, baseline ECG or method-comparison reports that did not mention those concepts may have been missed. The strategy was not retrospectively altered without a rerun; this sensitivity limitation and the final search date are reported explicitly.

### 2.5. Study Selection Process

Search records were managed in Covidence (web-based software; Veritas Health Innovation, Melbourne, Australia; https://www.covidence.org). Two reviewers independently assessed each assigned record at title/abstract and full-text stages.

Duplicate records were identified in Covidence and manually checked. The same eligibility framework was used at both screening stages.

Disagreements were resolved by discussion and criterion-by-criterion review; a reviewer who had not made the original pair of decisions adjudicated unresolved cases.

At title/abstract screening, raw agreement ranged from 0.455 to 0.857 and Cohen’s κ ranged from −0.077 to 0.137. The initial screen favoured retrieval when abstracts were unclear, and reviewers differed mainly in how broadly they interpreted heart rate, anaesthesia, haemodynamic and structural reports. These disagreements were resolved before full-text assessment.

Twenty-two reports underwent full-text assessment. Aggregate reasons were preserved in the screening record: six reports had an ineligible design and one had eligibility that could not be confirmed. Supplementary Table S1 provides all retrievable citation-level information. The complete citation-level log for the remaining archived exclusions was not available to the authors during this revision and has not been reconstructed by guessing.

The overall selection process is summarised in the PRISMA 2020 flow diagram (Figure 1).

### 2.6. Data Extraction

Two reviewer teams independently extracted study identification, design, setting, sample size, age, biological sex, health status, exposure, comparator, lead configuration, electrode placement, animal position, recording duration, ECG intervals, waveform features, rhythm or conduction abnormalities, heart rate variability, key findings, and methodological limitations. Direct ECG or electrical telemetry outcomes were recorded separately from contextual cardiovascular outcomes. Discrepancies were resolved by discussion and, when necessary, adjudication by a reviewer who had not made the original pair of decisions. The extraction framework and study-level evidence summary are provided in Supplementary Table S2.

### 2.7. Risk of Bias and Methodological Quality Assessment

The Systematic Review Centre for Laboratory Animal Experimentation (SYRCLE) risk-of-bias tool [14] was applied to the 11 prospective experimental studies. Two reviewer teams independently judged ten domains: sequence generation, baseline characteristics, allocation concealment, random housing, caregiver/investigator blinding, random outcome assessment, outcome-assessor blinding, incomplete outcome data, selective reporting, and other bias. Each domain was rated low, high, or unclear risk; no unsupported composite score was calculated. Disagreements were resolved by consensus or adjudication. The tool was not applied to cross-sectional or retrospective studies because several intervention-specific domains were not applicable; those designs were appraised narratively.

For observational studies, the narrative appraisal considered participant selection, clarity of eligibility criteria, completeness of ECG or cardiovascular methods, handling of missing data, and consistency between stated methods and reported outcomes. Detailed experimental-study judgments are provided in Supplementary Table S3.

### 2.8. Data Synthesis

Formal meta-analysis was inappropriate because study design, age, health status, exposure, lead configuration, animal position, recording duration, sampling, and outcome reporting differed substantially. Direct ECG/electrical telemetry evidence and contextual cardiovascular evidence were therefore synthesised separately using a structured narrative approach.

Reported means, ranges, standard deviations, and time-course values were reproduced when available. No pooled effect measure or new inferential statistical analysis was performed. Interval values were standardised to milliseconds for presentation; NR denotes not reported and NA denotes not applicable.

### 2.9. Subgroup and Sensitivity Analyses

Formal subgroup or sensitivity analyses were not conducted because the evidence was sparse and no quantitative synthesis was performed. Descriptive comparisons were made only when studies reported sufficiently comparable direct ECG outcomes. No interspecies comparison was possible because no eligible *Camelus bactrianus* study was identified.

### 2.10. Assessment of Publication Bias

Funnel plots and statistical tests for publication bias were not appropriate without a pooled analysis and with only 15 studies. The possibility of missing results was considered qualitatively in relation to small samples, incomplete methods, selective outcome reporting, the discoverability of older camel research, and the sensitivity limitation of the executed search.

## 3. Results

### 3.1. Study Selection

The database and supplementary searches identified 68 records: 57 from electronic databases (Scopus, *n* = 33; Web of Science, *n* = 11; Embase, *n* = 8; PubMed/MEDLINE, *n* = 5) and 11 from other sources. After 19 duplicates were removed, 49 records underwent title/abstract screening.

Twenty-two reports were assessed in full. Seven were excluded: six because of ineligible study designs, including the comparative review removed during verification, and one because eligibility could not be confirmed from the available information. Supplementary Table S1 gives all retrievable citation-level information and transparently identifies the archived records for which individual citations could not be recovered.

Fifteen primary studies were included in the qualitative synthesis. No studies were ongoing or awaiting classification.

### 3.2. Inter-Rater Agreement

At title/abstract screening, raw agreement ranged from 0.455 to 0.857 and Cohen’s κ ranged from −0.077 to 0.137. Cohen’s κ estimates agreement beyond chance. The initial screen deliberately favoured retrieval when abstracts were ambiguous, and disagreements most often concerned heart-rate-only or haemodynamic reports, structural or echocardiographic papers, and records with unclear design. Small pair-specific samples and imbalanced include/exclude decisions increased expected chance agreement, which can produce a low κ despite relatively high raw agreement.

Agreement improved at full-text screening (raw agreement 0.750–0.824; κ 0.463–0.500). All disagreements were resolved by consensus or adjudication. Table 1 presents the pair-specific denominators and reconstructable agreement data.

### 3.3. Characteristics of Included Studies

The 15 included primary studies were published from 1980 to 2025 and all involved *Camelus dromedarius*. Three were conducted in Saudi Arabia, two in Egypt, two in India, and eight did not clearly report country. Numerical sample sizes ranged from 5 to 93 animals (median 14); 12 studies enrolled 21 or fewer animals.

The evidence comprised seven non-randomised experiments, four randomised controlled trials, including two crossover trials, three cross-sectional studies, and one retrospective clinical series. Biological sex was explicitly reported for only a minority of cohorts: the detomidine study used five females, the furosemide study used 21 males, the 2023 dexmedetomidine study used six males, and the baseline echocardiographic cohort included both sexes; most other reports did not provide sex in a form that supported comparison. No study was designed or powered to estimate a sex effect.

Six studies provided direct ECG or electrical telemetry evidence. Nine reported contextual heart rate, Doppler, echocardiographic, or haemodynamic outcomes without complete ECG interval or rhythm data. Eleven studies involved a pharmacological intervention. Table 2 lists all included studies, study designs, reported sex, and evidence category.

### 3.4. Electrocardiographic Parameters and Findings

#### 3.4.1. Baseline Electrocardiographic Values

Across the direct ECG or electrical telemetry studies, reported resting heart rates ranged from approximately 33 to 89 beats/min. Pourjafar et al. [3] described an age-related decline from 89 beats/min in the youngest group to 48 beats/min in older camels, whereas Evans et al. [4] reported a resting value of 33 ± 2 beats/min before treadmill exercise. These values were not pooled because populations and recording methods differed.

PR interval data were available from few direct studies. Pourjafar et al. [3] reported 200–260 ms across age groups, whereas Samimi and Sanjarinejad [8] reported a control baseline value of 180 ms in calves. QRS and QT values were reported inconsistently. A relatively long PR interval may reflect autonomic tone or species-specific atrioventricular conduction, but none of the included studies tested this mechanism.

#### 3.4.2. Pharmacological Effects on ECG Parameters

Eleven studies examined pharmacological interventions, although several reported only heart rate or haemodynamic variables. Xylazine was associated with heart rates of 30–50 beats/min, a longer PR interval, sinus arrhythmia, wandering pacemaker activity, sinoatrial block, and first-degree atrioventricular block [5]. After detomidine, heart rate decreased from 60 ± 18 to 29 ± 6 beats/min at 5 min; QT and RR intervals increased, whereas the PR interval, wave amplitudes, and waveform configuration did not differ significantly from baseline [6].

The dexmedetomidine studies reported dose-related heart rate reductions but did not provide sufficiently complete ECG interval or morphology data for the direct electrophysiological table [18,19]. Medetomidine, propofol, lidocaine, and xylazine–ketamine reports were therefore treated as contextual cardiovascular evidence [9,11,15,17].

Chloral hydrate produced the most severe directly reported ECG disturbances: heart rate increased from 47 beats/min at baseline to 110 beats/min at 5 min and declined to 73 beats/min at 75 min; the PR interval changed from 250 to 140 and then 150 ms; and sinoatrial block, third-degree atrioventricular block, and ST-segment depression were observed [7]. These are source-study observations; the mechanism was not tested.

In the furosemide trial, clinical and ECG measurements were obtained at baseline and 2, 24, and 48 h after two intravenous doses. No statistically significant change in ECG indices was reported in the control, 2.5 mg/kg, or 5 mg/kg groups [8]. Table 3 presents baseline and follow-up values separately for every direct study for which a comparison was available.

#### 3.4.3. Exercise-Induced Changes

During incremental treadmill exercise, heart rate increased from 33 ± 2 beats/min at rest to 147 ± 4 beats/min at 8 m/s [4]. No arrhythmia or conduction disturbance was reported, but the cohort included only six camels and the electrical-recording details were limited; therefore, the study cannot establish absence of exercise-associated arrhythmia.

#### 3.4.4. Arrhythmias and Conduction Abnormalities

Rhythm or conduction abnormalities were reported mainly during pharmacological interventions. Xylazine was associated with sinus arrhythmia, wandering pacemaker activity, sinoatrial block, and first-degree atrioventricular block [5]; detomidine was associated with sinus arrhythmia and bradycardia in all five animals from 3 to 60 min [6]; and chloral hydrate was associated with sinoatrial block, third-degree atrioventricular block, and ST-segment depression [7]. Baseline and exercise reports did not describe arrhythmias, but absence of reporting is not evidence that continuous monitoring excluded an event. The separate atrial-fibrillation case remains contextual because case reports were excluded [12].

### 3.5. Supporting Cardiovascular and Structural Context

Nine included studies contributed contextual cardiovascular information, including clinical heart rate, respiratory rate, blood pressure, cardiac output, and Doppler or echocardiographic measurements [9,10,11,15,16,17,18,19,20]. Because they did not provide complete ECG intervals, waveforms, or rhythm assessment, they were not used to derive ECG values, arrhythmia frequency, or reference limits.

The developmental review by Marwa-Babiker [21] was excluded because it did not provide original primary data eligible for the systematic synthesis. It is cited only to explain that anatomy and histology can inform hypotheses about the conduction system; structural description is not a substitute for direct ECG or electrical telemetry evidence.

### 3.6. Methodological Observations

Ethical approval was explicitly reported in 6 of 15 studies (40%). Five described a base–apex ECG configuration, whereas several omitted lead placement, electrode type, animal position, recording duration, sampling rate, filtering, or beat-selection procedures. Sex reporting was incomplete, and no study compared male and female camels. These omissions reduce reproducibility and limit clinical interpretation.

### 3.7. Quality Assessment of Included Studies

Among the 11 prospective experimental studies assessed with SYRCLE, sequence generation was rated low-risk in 4 studies and allocation concealment was high-risk in 4 and unclear in 7. Random housing, caregiver blinding, and random outcome assessment were unclear in all 11. Outcome-assessor blinding was low-risk in 3, high-risk in 2, and unclear in 6; incomplete outcome data were low-risk in 10; and selective reporting was low-risk in all 11. These domain-level weaknesses reduce confidence in causal interpretations, particularly for drug-associated changes. Detailed judgments are provided in Supplementary Table S3.

## 4. Discussion

This review separates six studies with direct ECG or electrical telemetry outcomes from nine studies that reported only heart rate, Doppler, echocardiographic, or haemodynamic outcomes. The direct studies support the descriptive findings on ECG intervals and rhythm, whereas the contextual studies inform cardiovascular response only and are not used to infer waveform abnormalities, arrhythmia frequency, or reference intervals [3,4,5,6,7,8,9,10,11,15,16,17,18,19,20].

Low resting heart rates and PR intervals of approximately 180–260 ms recurred in the direct studies [3,4,8]. Age and acquisition method appear to influence these measurements. A relatively long PR interval could reflect autonomic tone or species-specific atrioventricular conduction, but the available studies did not test the underlying mechanism and do not justify a diagnostic threshold.

The clearest pharmacological pattern was bradycardia after α2-adrenoceptor agonists. Xylazine and detomidine studies also reported sinus arrhythmia and sinoatrial or atrioventricular conduction changes [5,6]. Contextual studies based only on clinical heart rate or haemodynamics support cardiovascular sensitivity to sedation but cannot be interpreted as equivalent ECG evidence [9,11,15,16,17,18,19].

Chloral hydrate was directly associated with tachycardia, PR interval change, sinoatrial block, third-degree atrioventricular block, and ST-segment depression [7]. Autonomic compensation, direct myocardial or conduction-system depression, and metabolic disturbance are possible explanations, but they remain hypotheses because the source study did not measure the mechanism.

The exercise study demonstrated a large heart rate reserve [4], while the excluded atrial-fibrillation case confirms that clinically important arrhythmia can occur [12]. Neither report permits estimation of arrhythmia prevalence. Developmental and echocardiographic literature can inform structural hypotheses, but it should not be counted as direct electrophysiological evidence [10,11,20,21].

Confidence in the principal findings is limited. Most studies enrolled few animals, sex was often unreported, and lead placement, animal position, recording duration, filtering, and beat selection were inconsistently described. Randomisation, allocation concealment, housing, and blinding were also commonly unclear. These limitations precluded quantitative pooling, sex-specific inference, causal conclusions, and validated reference intervals.

The search strategy is an additional major limitation. Although the librarian verified that the saved strategy contained the main ECG and conduction terms, the mandatory exposure/context block may have missed baseline ECG or method-comparison reports that did not mention dehydration, stress, exercise, anaesthesia, or sedation. Furthermore, no new search was conducted after 22 December 2025. The review should therefore be interpreted as a synthesis of the retrieved evidence up to that cutoff, not as assurance that all eligible camel ECG studies were captured.

The available evidence supports cautious awareness of drug-associated rhythm and conduction changes, not treatment recommendations, screening thresholds, or definitive reference intervals. Future studies should use standardised lead configurations and animal positions, digital acquisition with reported sampling and filtering, longer rhythm monitoring, larger sex-diverse cohorts, prespecified outcomes, and transparent risk-control procedures. No inference can be made for *Camelus bactrianus*.

## 5. Conclusions

Direct ECG and electrical telemetry studies in *Camelus dromedarius* describe low resting heart rates, PR intervals of approximately 180–260 ms, and drug-associated rhythm or conduction abnormalities; supporting heart rate and haemodynamic studies provide context but do not establish ECG effects. Confidence is limited by few studies, small samples, heterogeneous acquisition methods, incomplete sex reporting, methodological bias, and the sensitivity and date limitations of the search. The reported values are preliminary descriptors rather than validated reference intervals, and no conclusion can be drawn for *Camelus bactrianus*. Standardised, adequately powered ECG studies with longer rhythm monitoring and sex-specific reporting are required.

## Figures and Tables

**Figure 1:**
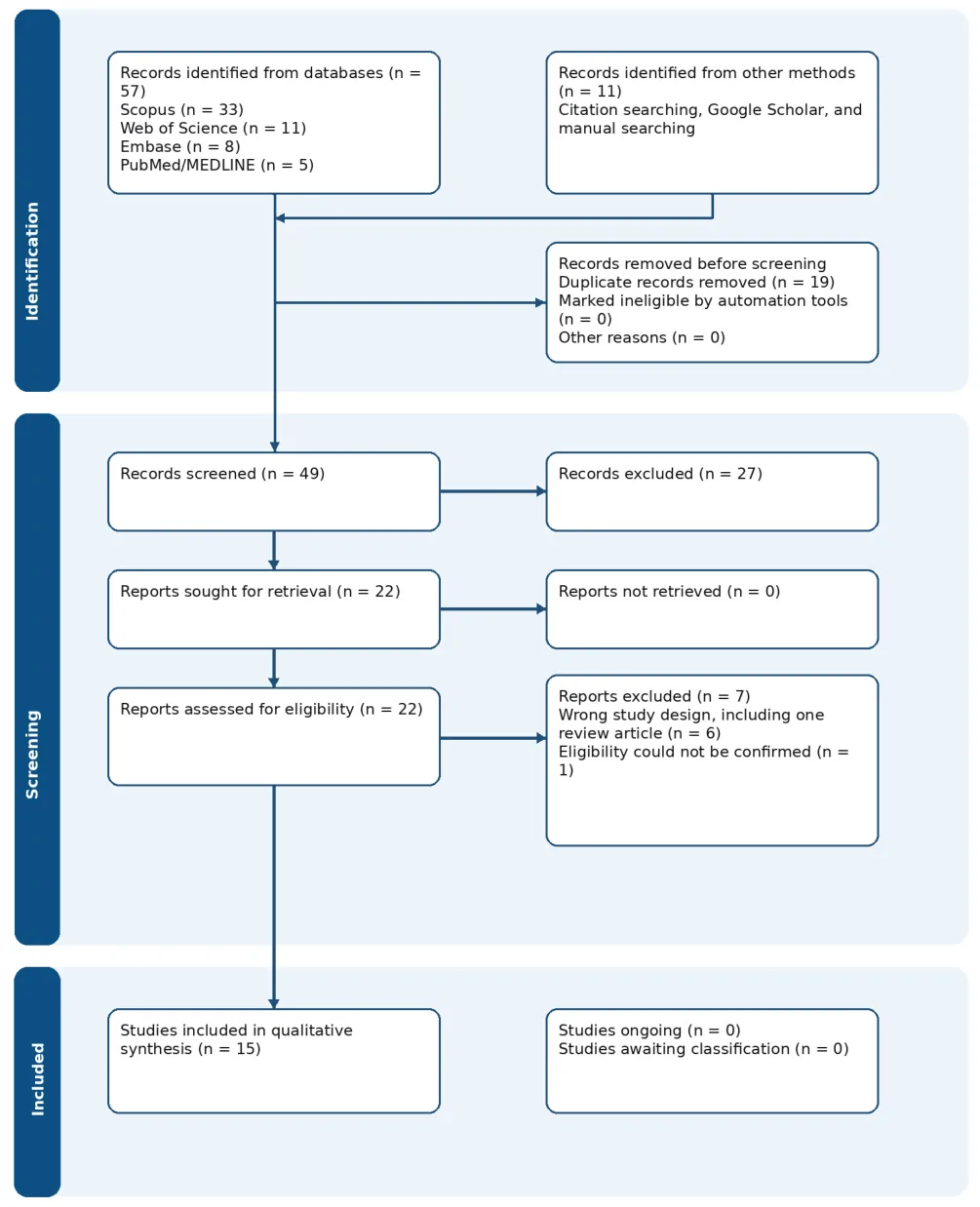
Preferred Reporting Items for Systematic Reviews and Meta-Analyses (PRISMA) 2020 flow diagram showing study identification, screening, eligibility assessment, and inclusion.

**Table 1:** Inter-rater agreement at title/abstract and full-text screening.

Stage	Reviewer Pair	Both Include	Both Exclude	A Include/B Exclude	A Exclude/B Include	*N*	Raw Agreement	Cohen’s κ
Title/abstract	FH vs. JA	2	3	5	1	11	0.455	0.029
Title/abstract	JA vs. MA/AA	0	12	1	1	14	0.857	−0.077
Title/abstract	FH vs. MA/AA	2	10	11	0	23	0.522	0.137
Full text	FH vs. MA/AA	2	1	1	0	4	0.750	0.500
Full text	JA vs. MA/AA	12	2	1	2	17	0.824	0.463

FH = Frank C. Howarth; JA = Jawaher Abdulhakim; MA/AA = Mariam Alnuaimi and Alyazia Alshamsi; *N* = number of records assessed by each reviewer pair; κ = Cohen’s kappa.

**Table 2:** Characteristics and evidence category of the 15 included primary studies.

Study	Country	Design	Species	*N*	Age; Sex	Health	Evidence Category and Exposure	ECG/HR Method
Peshin (1980) [5]	NR	Non-RCT	*C. dromedarius*	25	7–16 y; NR	Healthy/surgical	Direct ECG: Xylazine (0.4 mg/kg IM)	Base–apex surface ECG
Sharma (1983) [7]	NR	Non-RCT	*C. dromedarius*	6	7–16 y; NR	Healthy	Direct ECG: Chloral hydrate (100 mg/kg IV)	Base–apex surface ECG
Evans et al. (1994) [4]	NR	Non-RCT	*C. dromedarius*	6	7–12 y; NR	Healthy	Direct electrical telemetry: treadmill exercise	Telemetry; recording detail limited
Pourjafar et al. (2011) [3]	NR	Cross-sectional	*C. dromedarius*	93	<6 mo–18 y; NR	Healthy	Direct ECG: Baseline/restraint	Base–apex surface ECG
Samimi and Azari (2017) [6]	NR	Non-RCT	*C. dromedarius*	5	2 y; female	Healthy	Direct ECG: Detomidine (50 µg/kg IV)	Base–apex surface ECG
Samimi and Sanjarinejad (2021) [8]	NR	RCT	*C. dromedarius*	21	5 ± 1 mo; male	Healthy	Direct ECG: Furosemide (2.5 and 5 mg/kg IV)	Base–apex surface ECG
Al-Mubarak (2008) [9]	KSA	Non-RCT	*C. dromedarius*	14	6–9 mo; NR	Healthy	Contextual HR/haemodynamics: Propofol TIVA	HR method NR
Al-Mubarak et al. (2008) [15]	KSA	Retrospective	*C. dromedarius*	55	6.7 ± 2.6 y; NR	Diseased	Contextual HR/haemodynamics: Xylazine–ketamine TIVA	Clinical HR; ECG detail NR
Dehghan et al. (2011) [10]	NR	Cross-sectional	*C. dromedarius*	20	1 y; both sexes	Healthy	Contextual HR/echocardiography: Baseline	Echocardiography; clinical HR
Shekidef and Saleh (2011) [16]	Egypt	RCT	*C. dromedarius*	6	Adult; NR	Healthy	Contextual HR: Epidural ketamine/xylazine or medetomidine	Clinical HR; ECG detail NR
Almubarak (2013) [17]	KSA	Non-RCT	*C. dromedarius*	7	4.8 y; NR	Healthy	Contextual HR: Lidocaine (3 mg/kg IV)	Clinical HR; ECG detail NR
Masoudifard et al. (2020) [11]	NR	Non-RCT	*C. dromedarius*	20	6–8 mo; NR	Healthy	Contextual HR/haemodynamics: Medetomidine or xylazine	Pulsed-wave Doppler; HR
Nath et al. (2023) [18]	India	RCT crossover	*C. dromedarius*	6	4–5 y; male	Healthy	Contextual HR: Dexmedetomidine (2.5 or 4 µg/kg IV)	Clinical HR; ECG detail NR
Nath et al. (2024) [19]	India	RCT crossover	*C. dromedarius*	6	4–5 y; NR	Healthy	Contextual HR: Dexmedetomidine/butorphanol/ketamine	Clinical HR; ECG detail NR
El-Sayed et al. (2025) [20]	Egypt	Cross-sectional	*C. dromedarius*	20	17.1 ± 1.5 y; NR	Healthy	Contextual HR/echocardiography: Baseline	Doppler echocardiography; clinical HR

ECG = electrocardiography; HR = heart rate; IM = intramuscular; IV = intravenous; KSA = Kingdom of Saudi Arabia; *N* = sample size; mo = months; y = years; RCT = randomised controlled trial; Non-RCT = non-randomised experimental study; TIVA = total intravenous anaesthesia; NR = not reported. Supporting studies were not used to derive ECG interval estimates or arrhythmia frequency.

**Table 3:** Baseline and post-intervention or exercise values from studies with direct ECG or electrical telemetry outcomes.

Study	Exposure/Comparison	Baseline HR (beats/min)	Post-Intervention/Peak HR (beats/min)	ECG Interval Comparison	Timing	Rhythm/Conduction Findings
Pourjafar et al. (2011) [3]	Cross-sectional baseline by age group	48–89	NA	PR 200–260 ms; QRS/QT NR	Age groups <6 mo to 16–18 y	None reported
Peshin (1980) [5]	Xylazine 0.4 mg/kg IM	NR	30–50	PR increased; QRS and QT reported as unchanged	Post-administration; exact sampling times not available in review extraction	Sinus arrhythmia; wandering pacemaker; SA block; first-degree AV block
Sharma (1983) [7]	Chloral hydrate 100 mg/kg IV	47	110 at 5 min; 73 at 75 min	PR 250 → 140 ms at 5 min → 150 ms at 75 min; QRS/QT NR	5 and 75 min	SA block; third-degree AV block; ST depression
Samimi and Azari (2017) [6]	Detomidine 50 µg/kg IV	60 ± 18	29 ± 6 at 5 min	QT 370 ± 60 → 440 ± 30 ms at 10 min; PR not significantly changed	3–90 min; rhythm changes reported from 3 to 60 min	Sinus arrhythmia and bradycardia in 5/5 camels
Samimi and Sanjarinejad (2021) [8]	Two IV doses of furosemide (2.5 or 5 mg/kg; 12 h apart)	Control 80 ± 8; low dose 80 ± 11; high dose 79 ± 9	No significant HR or ECG-index change in any group	Control baseline PR 180 ms, QRS 100 ms, QT 380 ms; no significant change reported	2, 24, and 48 h	None reported
Evans et al. (1994) [4]	Incremental treadmill exercise	33 ± 2	147 ± 4 at 8 m/s	PR/QRS/QT NR	Peak at 8 m/s	None reported

ECG = electrocardiography; HR = heart rate; IM = intramuscular; IV = intravenous; PR = PR interval; QRS = QRS complex duration; QT = QT interval; SA = sinoatrial; AV = atrioventricular; NR = not reported; NA = not applicable. Interval values are in milliseconds. ‘None reported’ indicates that the source paper did not report an event; it does not establish that continuous monitoring excluded an event.

## Data Availability

The original contributions presented in the study are included in the article/Supplementary Materials. Further inquiries can be directed to the corresponding author.

## Supplementary Materials

The following supporting information can be downloaded at https://www.mdpi.com/article/10.3390/biology15191711/s1: PRISMA 2020 checklist; Supplementary File S1: complete original database search strategies; Supplementary Table S1: available full-text exclusion information; Supplementary Table S2: extraction framework and study-level evidence summary; Supplementary Table S3: SYRCLE Risk-of-Bias Judgments.

## Abbreviations

The following abbreviations are used in this manuscript:

**Abbreviation**	**Meaning**
AV	atrioventricular
ECG	electrocardiography/electrocardiogram
HR	heart rate
HRV	heart rate variability
IM	intramuscular
IV	intravenous
KSA	Kingdom of Saudi Arabia
NA	not applicable
Non-RCT	non-randomised experimental study
NR	not reported
PECO	Population, Exposure, Comparator and Outcome
PRISMA	Preferred Reporting Items for Systematic Reviews and Meta-Analyses
PROSPERO	International Prospective Register of Systematic Reviews
QTc	corrected QT interval
RCT	randomised controlled trial
SA	sinoatrial
SYRCLE	Systematic Review Centre for Laboratory Animal Experimentation
TIVA	total intravenous anaesthesia
