# Supplementary material for: Electrophysiology of the Camel Heart: A Systematic Review of Evidence from Camelus dromedarius
Source: Biology (Basel). 2026 Sep 26;15(19):1711. doi: 10.3390/biology15191711 (PMC13663444; doi:10.3390/biology15191711)
Supplement: Supplementary file 1 [file biology-15-01711-s001.zip › biology-4509539_05_Special_Issue_PRISMA_2020_Checklist.pdf]

PRISMA 2020 Checklist

Manuscript: Electrophysiology of the camel heart: A systematic review of evidence from Camelus dromedarius

Item	Section/topic	PRISMA 2020 reporting item	Location in revised manuscript / explanation	Status
**1**	Title	Identify the report as a systematic review.	Title	Reported
**2**	Abstract	Provide a structured summary of objectives, methods, results, limitations, conclusions, and registration.	Structured Abstract; registration included	Reported
**3**	Rationale	Describe the rationale in the context of existing knowledge.	Introduction	Reported
**4**	Objectives	State the review objective or question explicitly.	Introduction, final paragraph	Reported
**5**	Eligibility criteria	Specify inclusion and exclusion criteria and grouping for synthesis.	Methods, Section 2.2	Reported
**6**	Information sources	Specify databases and other sources and the date each was last searched.	Methods, Section 2.3; Supplementary File S1	Reported
**7**	Search strategy	Present complete search strategies for all databases, including filters and limits.	Supplementary File S1	Reported
**8**	Selection process	Describe how studies were judged eligible, number of reviewers, independence, and resolution of disagreement.	Methods, Section 2.5	Reported
**9**	Data collection	Describe data collection methods, number of reviewers, independence, and disagreement handling.	Methods, Section 2.6; Supplementary Table S3	Reported
**10a**	Data items: outcomes	List and define all outcomes sought and whether all compatible results were collected.	Methods, Section 2.2.3; Supplementary Table S3	Reported
**10b**	Data items: other variables	List and define other variables sought and assumptions regarding missing information.	Methods, Section 2.6; Supplementary Table S3	Reported
**11**	Risk of bias	Specify the tool, domains, reviewers, independence, and resolution process.	Methods, Section 2.7; Supplementary Table S4	Reported
**12**	Effect measures	Specify the effect measure for each outcome.	Methods, Section 2.8; no pooled effect measure	Not applicable / explained
**13a**	Eligibility for syntheses	Describe how studies were assigned to each synthesis.	Methods, Sections 2.2 and 2.8	Reported
**13b**	Data preparation	Describe preparation of data for presentation or synthesis.	Methods, Section 2.6	Reported
**13c**	Tabulation and display	Describe methods used to tabulate or visually display results.	Methods, Section 2.8; Tables 1-3; Figures 1-2	Reported
**13d**	Synthesis methods	Describe methods used to synthesize results and justify choices.	Methods, Section 2.8	Reported
**13e**	Exploration of heterogeneity	Describe methods used to explore possible causes of heterogeneity.	Methods, Section 2.9	Reported descriptively
**13f**	Sensitivity analyses	Describe planned sensitivity analyses.	Methods, Section 2.9	Not applicable / explained
**14**	Reporting bias methods	Describe methods to assess risk of bias due to missing results.	Methods, Section 2.10	Reported qualitatively
**15**	Certainty assessment	Describe methods to assess certainty or confidence in the body of evidence.	Methods, Section 2.7; no formal certainty framework	Not undertaken / explained
**16a**	Study selection results	Report results from records identified through studies included, ideally with a flow diagram.	Results, Section 3.1; Figure 2	Reported

Item	Section/topic	PRISMA 2020 reporting item	Location in revised manuscript / explanation	Status
**16b**	Excluded reports	Cite reports that appeared eligible but were excluded and explain why.	Results, Section 3.1; Supplementary Table S2	Partially reported: aggregate reasons and retrievable citation supplied; complete archived citation-level log unavailable
**17**	Study characteristics	Cite each included study and present its characteristics.	Results, Section 3.3; Table 2; Supplementary Table S3	Reported
**18**	Risk of bias results	Present risk-of-bias assessments for each included study.	Results, Section 3.7; Supplementary Table S4	Reported
**19**	Individual study results	For each outcome, present summary statistics and effect estimates where applicable.	Results, Sections 3.4-3.5; Tables 2-3	Reported
**20a**	Synthesis summary	Summarize contributing-study characteristics and risk of bias for each synthesis.	Results, Sections 3.3 and 3.7	Reported
**20b**	Statistical syntheses	Present results of all statistical syntheses.	No statistical synthesis; Methods, Section 2.8	Not applicable / explained
**20c**	Causes of heterogeneity	Present results of investigations of possible causes of heterogeneity.	Results and Discussion	Reported descriptively
**20d**	Sensitivity analyses results	Present results of sensitivity analyses.	No sensitivity analysis; Methods, Section 2.9	Not applicable / explained
**21**	Reporting bias results	Present assessments of risk of bias due to missing results.	Methods, Section 2.10; Discussion	Reported qualitatively
**22**	Certainty results	Present certainty or confidence in the body of evidence.	Discussion; no formal certainty framework	Not undertaken / explained
**23a**	Interpretation	Provide a general interpretation in the context of other evidence.	Discussion	Reported
**23b**	Limitations of evidence	Discuss limitations of the included evidence.	Discussion	Reported
**23c**	Limitations of review process	Discuss limitations of the review methods.	Discussion, including search limitation	Reported
**23d**	Implications	Discuss implications for practice and future research.	Discussion and Conclusions	Reported
**24a**	Registration	Provide registration information, including register and number.	Abstract and Methods, Section 2.1	Reported
**24b**	Protocol access	State where the protocol can be accessed.	Methods, Section 2.1; PROSPERO CRD420251169514	Reported
**24c**	Protocol amendments	Describe and explain amendments or state none.	Methods, Sections 2.1 and 2.2.5; no protocol amendments	Reported
**25**	Support	Describe financial and non-financial support and the role of funders.	Funding statement	Reported
**26**	Competing interests	Declare competing interests.	Conflicts of Interest statement	Reported
**27**	Availability of materials	State which data, code, forms, or other materials are publicly available and where.	Data Availability Statement; Supplementary Materials	Reported with stated archive limitation

Locations are given by manuscript section, table or supplementary item because final pagination is generated by the journal system.
