# Supplementary material for: Electrophysiology of the Camel Heart: A Systematic Review of Evidence from Camelus dromedarius
Source: Biology (Basel). 2026 Sep 26;15(19):1711. doi: 10.3390/biology15191711 (PMC13663444; doi:10.3390/biology15191711)
Supplement: Supplementary file 1 [file biology-15-01711-s001.zip › biology-4509539_06_Special_Issue_Supplementary_Materials.pdf]

Supplementary Materials

Supplementary File S1. Complete Original Database Search Strategies

1. Search Record Summary

Source	Records in screening dataset	Notes
PubMed/MEDLINE	5	English-language limit applied.
Scopus	33	Screening count used in the PRISMA flow.
Embase	8	Article and English-language limits applied.
Web of Science	11	Article and English-language limits applied.
**Electronic databases**	**57**	Subtotal before deduplication.
Other methods	11	Google Scholar, citation tracking, manual journal searching, and subject-expert input.
**Total records**	**68**	Total before removal of 19 duplicates.

2. PubMed/MEDLINE

Records in screening dataset: n = 5 | Limit: English

```
(((((((((((((Camelus[MeSH Terms]) OR ("camel* dromedary"[Title/Abstract])) OR (camel[Title/Abstract])) OR (camels[Title/Abstract])) OR (dromedarius[Title/Abstract])) OR ("camelus bactrian*"[Title/Abstract])) OR
("dromedar*"[Title/Abstract])) OR ("hybrid camel*"[Title/Abstract])) OR ("one humped camel*"[Title/Abstract])) OR ("two humped camel*"[Title/Abstract]))) AND
((((((((((((((((((((((((((((("electrocardiograph*"[Title/Abstract]) OR ("ECG"[Title/Abstract])) OR ("EKG"[Title/Abstract])) OR ("heart rate variability"[Title/Abstract])) OR ("electrophysiol*"[Title/Abstract])) OR ("ion
channel"[Title/Abstract])) OR ("conduction system"[Title/Abstract])) OR ("arrhythmia*"[Title/Abstract])) OR ("PR interval"[Title/Abstract])) OR ("QT"[Title/Abstract])) OR ("P wave"[Title/Abstract])) OR
("QRS"[Title/Abstract])) OR ("T wave morphology"[Title/Abstract])) OR ("intervals"[Title/Abstract])) OR ("biopotential"[Title/Abstract])) OR ("electrophysiological phenomena"[Title/Abstract])) OR ("electrophysiological
processes"[Title/Abstract])) OR ("cardiac conduction"[Title/Abstract])) OR ("cardiac electrophysiology"[Title/Abstract])) OR ("action potential"[Title/Abstract])) OR ("electrical activit*"[Title/Abstract])) OR
("electrophysiology"[MeSH Terms])) OR ("axis"[Title/Abstract])) OR (Electrocardiogram[Title/Abstract])) OR ("Cardiac remodeling"[Title/Abstract])) OR ("Autonomic regulation"[Title/Abstract])) OR
(biopotential[Title/Abstract])) OR ("autonomic regulation"[Title/Abstract]) OR (("Cardiac"[Title/Abstract] AND "ion channels")))) AND (((((((((((((((((heart[MeSH Terms]) OR (cardiovascular system[MeSH Terms])) OR
(heart*[Title/Abstract])) OR (cardiac[Title/Abstract])) OR ("myocard*"[Title/Abstract])) OR (cardiomyopathy[Title/Abstract])) OR (cardiovascular[Title/Abstract])) OR ("ventricle"[Title/Abstract])) OR
(atrium[Title/Abstract])) OR (Atria[Title/Abstract])) OR ("Sinoatrial Node"[Title/Abstract])) OR ("Atrioventricular Node"[Title/Abstract])) OR ("Purkinje Fiber*"[Title/Abstract])) OR (Cardiomyocytes[Title/Abstract]))
OR (Myocytes[Title/Abstract])) OR ("Cardiac Muscle"[Title/Abstract])) OR ("Gap Junctions"[Title/Abstract])))) AND ((((((((((dehydration[MeSH Terms]) OR (dehydration[Title/Abstract])) OR ("heat stress"[Title/Abstract]))
OR (temperature[Title/Abstract])) OR ("environmental stress"[Title/Abstract])) OR (stress*[Title/Abstract])) OR (workload[Title/Abstract])) OR (exercise[Title/Abstract])) OR (anesthesia[Title/Abstract])) OR
(sedation[Title/Abstract])) Filters: English
```

3. Scopus

Records in screening dataset: n = 33 | Limits: Article; English

(((TITLE-ABS-KEY ("camelus dromedarius") OR TITLE-ABS-KEY ("Bactrian* Camel*") OR TITLE-ABS-KEY ("Camel*, Bactrian*") OR TITLE-ABS-KEY ("Bactrian* Camel*") OR TITLE-ABS-KEY ("Camel*, Bactrian*") OR TITLE-ABS-KEY (Camelus) OR TITLE-ABS-KEY ("camel* dromedary") OR TITLE-ABS-KEY (camel) OR TITLE-ABS-KEY (camels) OR TITLE-ABS-KEY (dromedarius) OR TITLE-ABS-KEY ("camelus bactrian*") OR TITLE-ABS-KEY (dromedar*) OR TITLE-ABS-KEY ("hybrid camel*") OR TITLE-ABS-KEY ("one humped camel*") OR TITLE-ABS-KEY ("two humped camel*"))) AND ((TITLE-ABS-KEY ("electrocardiograph*") OR TITLE-ABS-KEY ("ECG") OR TITLE-ABS-KEY ("EKG") OR TITLE-ABS-KEY ("heart rate variability") OR TITLE-ABS-KEY ("electrophysiolog*") OR TITLE-ABS-KEY ("action potential") OR TITLE-ABS-KEY ("ion channel") OR TITLE-ABS-KEY ("conduction system") OR TITLE-ABS-KEY ("arrhythmia*") OR TITLE-ABS-KEY ("PR interval") OR TITLE-ABS-KEY ("QT") OR TITLE-ABS-KEY ("P wave") OR TITLE-ABS-KEY ("QRS") OR TITLE-ABS-KEY ("T wave morphology") OR TITLE-ABS-KEY ("intervals") OR TITLE-ABS-KEY ("biopotential") OR TITLE-ABS-KEY ("electrophysiological phenomena") OR TITLE-ABS-KEY ("electrophysiological processes") OR TITLE-ABS-KEY ("electrophysiology") OR TITLE-ABS-KEY ("electrophysiol*") OR TITLE-ABS-KEY ("cardiac conduction") OR TITLE-ABS-KEY ("cardiac electrophysiology") OR TITLE-ABS-KEY ("action potential") OR TITLE-ABS-KEY ("electrical activit*") OR TITLE-ABS-KEY ("axis") OR TITLE-ABS-KEY (Electrocardiogram*) OR TITLE-ABS-KEY ("Cardiac remodeling") OR TITLE-ABS-KEY ("Autonomic regulation") OR TITLE-ABS-KEY (("Cardiac" AND "ion channels")))) AND ((TITLE-ABS-KEY ("Cardiovascular Systems") OR TITLE-ABS-KEY ("Circulatory System*") OR TITLE-ABS-KEY (heart*) OR TITLE-ABS-KEY (cardic) OR TITLE-ABS-KEY ("myocard*") OR TITLE-ABS-KEY (cardiomyopath*) OR TITLE-ABS-KEY (cardiovascular) OR TITLE-ABS-KEY (ventricle) OR TITLE-ABS-KEY (atrium) OR TITLE-ABS-KEY (Atria) OR TITLE-ABS-KEY ("Sinoatrial Node") OR TITLE-ABS-KEY ("Atrioventricular Node") OR TITLE-ABS-KEY ("Purkinje Fiber*") OR TITLE-ABS-KEY (Cardiomyocytes) OR TITLE-ABS-KEY (Myocytes) OR TITLE-ABS-KEY ("Cardiac Muscle") OR TITLE-ABS-KEY ("Gap Junctions")))) AND ((TITLE-ABS-KEY (dehydration) OR TITLE-ABS-KEY ("heat stress") OR TITLE-ABS-KEY (temperature) OR TITLE-ABS-KEY ("environmental stress") OR TITLE-ABS-KEY (stress*) OR TITLE-ABS-KEY (workload) OR TITLE-ABS-KEY (exercise) OR TITLE-ABS-KEY (anesthesia) OR TITLE-ABS-KEY (sedation) OR TITLE-ABS-KEY ("Water Stress") OR TITLE-ABS-KEY ("Stress, Water"))) AND (LIMIT-TO (DOCTYPE , "ar")) AND (LIMIT-TO (LANGUAGE , "English"))

4. Embase

Records in screening dataset: n = 8 | Limits: Article; English

```
('camel'/exp OR 'camel* dromedary':ti,ab,kw OR 'camel':ti,ab,kw OR 'camels':ti,ab,kw OR 'dromedarius':ti,ab,kw OR 'camelus bactrian*':ti,ab,kw OR 'dromedar*':ti,ab,kw OR 'hybrid camel*':ti,ab,kw OR 'one humped camel*':ti,ab,kw OR 'two humped camel*':ti,ab,kw) AND ('electrocardiograph*':ti,ab,kw OR 'ecg':ti,ab,kw OR 'ekg':ti,ab,kw OR 'heart rate variability':ti,ab,kw OR 'electrophysiol*':ti,ab,kw OR 'ion channel':ti,ab,kw OR 'conduction system':ti,ab,kw OR 'arrhythmia*':ti,ab,kw OR 'pr interval':ti,ab,kw OR 'qt':ti,ab,kw OR 'p wave':ti,ab,kw OR 'qrs':ti,ab,kw OR 't wave morphology':ti,ab,kw OR 'intervals':ti,ab,kw OR 'electrophysiological phenomena':ti,ab,kw OR 'electrophysiological processes':ti,ab,kw OR 'cardiac conduction':ti,ab,kw OR 'cardiac electrophysiology':ti,ab,kw OR 'action potential':ti,ab,kw OR 'electrical activit*':ti,ab,kw OR 'electrophysiology'/exp OR 'axis':ti,ab,kw OR 'electrocardiogram':ti,ab,kw OR 'cardiac remodeling':ti,ab,kw OR 'biopotential':ti,ab,kw OR 'autonomic regulation':ti,ab,kw OR ('cardiac':ti,ab,kw AND 'ion channels')) AND ('heart'/exp OR 'cardiovascular system'/exp OR 'heart*':ti,ab,kw OR 'cardiac':ti,ab,kw OR 'myocard*':ti,ab,kw OR 'cardiomyopathy':ti,ab,kw OR 'cardiovascular':ti,ab,kw OR 'ventricle':ti,ab,kw OR 'atrium':ti,ab,kw OR 'atria':ti,ab,kw OR 'sinoatrial node':ti,ab,kw OR 'atrioventricular node':ti,ab,kw OR 'purkinje fiber*':ti,ab,kw OR 'cardiomyocytes':ti,ab,kw OR 'myocytes':ti,ab,kw OR 'cardiac muscle':ti,ab,kw OR 'gap junctions':ti,ab,kw) AND ('dehydration'/exp OR 'dehydration':ti,ab,kw OR 'heat stress':ti,ab,kw OR 'temperature':ti,ab,kw OR 'environmental stress':ti,ab,kw OR 'stress*':ti,ab,kw OR 'workload':ti,ab,kw OR 'exercise':ti,ab,kw OR 'anesthesia':ti,ab,kw OR 'sedation':ti,ab,kw) AND 'article'/it AND [english]/lim
```

5. Web of Science Core Collection

Records in screening dataset: n = 11 | Limits: Article; English

The saved Web of Science history was supplied as separate concept sets. For readability, the sets are reproduced below using consistent numbering; the terms are unchanged.

Set 1 - Camel population terms

```
TS=("Camelus dromedarius" OR "Bactrian* Camel*" OR "Camel*, Bactrian*" OR Camelus OR "camel* dromedary" OR camel OR camels OR dromedarius OR "camelus bactrian*" OR dromedar* OR "hybrid camel*" OR "one humped camel*" OR "two humped camel*")
```

Set 2 - ECG/electrophysiology terms

```
TS=("electrocardiograph*" OR ECG OR EKG OR "heart rate variability" OR "electrophysiolog*" OR "action potential" OR "ion channel" OR "conduction system" OR "arrhythmia*" OR "PR interval" OR QT OR "P wave" OR QRS OR "T wave morphology" OR intervals OR biopotential OR "electrophysiological phenomena" OR "electrophysiological processes" OR electrophysiology OR "cardiac conduction" OR "cardiac electrophysiology" OR "electrical activit*" OR axis OR Electrocardiogram* OR "Cardiac remodeling" OR "Autonomic regulation" OR (Cardiac AND "ion channels"))
```

Set 3 - Cardiac/cardiovascular terms

```
TS=("Cardiovascular Systems" OR "Circulatory System*" OR heart* OR cardic OR myocard* OR cardiomyopath* OR cardiovascular OR ventricle OR atrium OR atria OR "Sinoatrial Node" OR "Atrioventricular Node" OR "Purkinje Fiber*" OR Cardiomyocytes OR Myocytes OR "Cardiac Muscle" OR "Gap Junctions")
```

Set 4 - Exposure/context terms

```
TS=(dehydration OR "heat stress" OR temperature OR "environmental stress" OR stress* OR workload OR exercise OR anesthesia OR sedation OR "Water Stress" OR "Stress, Water")
```

Final combination

```
#1 AND #2 AND #3 AND #4; Document type: Article; Language: English.
```

6. Supplementary Searching

Google Scholar, backward citation tracking of included papers, manual searching of relevant journals, and subject-expert input were used to improve recall. These sources contributed 11 records to the screening dataset.

7. Search Reporting Note

This supplement provides the complete database-specific strategies actually used in the review. The final search date was 22 December 2025. After peer review, the research librarian rechecked the saved strategy and confirmed that PR interval, QT, QRS, heart rate variability, cardiac conduction, electrophysiology and arrhythmia terminology was represented. No new database search was conducted.

The search required an exposure/context concept block. Consequently, baseline ECG or method-comparison papers that did not mention dehydration, heat stress, exercise, anesthesia, sedation or related context terms could have been missed. This limitation is acknowledged in the revised manuscript; the executed search has not been retrospectively rewritten.

Supplementary Table S1. Available Full-Text Exclusion Information

The archived materials available for this revision retained aggregate exclusion counts but not the complete citation-level log. The authors provide every retrievable item below and have not invented missing citations.

Report/citation	Specific reason	Record status
Marwa-Babiker, A.M. Unique development of the heart of the dromedary camel: A comparative review. J. Camel Pract. Res. 2023, 30, 301-309.	Review article; ineligible study design.	Citation identifiable from the corrected included-study set.
Five full-text reports; individual citations not retained in the available archived exclusion record.	Ineligible study design (aggregate category recorded in Covidence).	Citation-level details unavailable during this revision.
One full-text report; individual citation not retained in the available archived exclusion record.	Eligibility could not be confirmed from the available full-text information.	Citation-level details unavailable during this revision.

Reporting limitation: PRISMA item 16b is therefore only partially fulfilled. The absence of the original citation-level exclusion log is disclosed in the manuscript and response letter.

Supplementary Table S2. Extraction Framework and Study-Level Evidence Summary

The extraction framework included study design, sample characteristics, biological sex when reported, exposure, ECG/telemetry method, direct electrophysiological outcomes, contextual cardiovascular outcomes and methodological limitations.

Study	Country	Design	Species	N	Age	Health	Evidence category and exposure	ECG/HR method
Peshin (1980) [5]	NR	Non-RCT	*C. dromedarius*	25	7–16 y	Healthy/surgical	Direct ECG: xylazine (0.4 mg/kg IM)	Base-apex surface ECG
Sharma (1983) [7]	NR	Non-RCT	*C. dromedarius*	6	7–16 y	Healthy	Direct ECG: chloral hydrate (100 mg/kg IV)	Base-apex surface ECG
Evans et al. (1994) [4]	NR	Non-RCT	*C. dromedarius*	6	7–12 y	Healthy	Direct telemetry: treadmill exercise	Telemetry; recording detail limited
Pourjafar et al. (2011) [3]	NR	Cross-sectional	*C. dromedarius*	93	<6 mo–18 y	Healthy	Direct ECG: baseline/restraint	Base-apex surface ECG
Samimi and Azari (2017) [6]	NR	Non-RCT	*C. dromedarius*	5	2 y	Healthy	Direct ECG: detomidine (50 µg/kg IV)	Base-apex surface ECG
Samimi and Sanjarinejad (2021) [8]	NR	RCT	*C. dromedarius*	21	5 ± 1 mo	Healthy	Direct ECG: furosemide (2.5 and 5 mg/kg IV)	Base-apex surface ECG
Al-Mubarak (2008) [9]	KSA	Non-RCT	*C. dromedarius*	14	6–9 mo	Healthy	Supporting HR/hemodynamics: propofol TIVA	HR method NR
Al-Mubarak et al. (2008) [15]	KSA	Retrospective	*C. dromedarius*	55	6.7 ± 2.6 y	Diseased	Supporting HR/hemodynamics: xylazine–ketamine TIVA	Clinical HR; ECG detail NR
Dehghan et al. (2011) [10]	NR	Cross-sectional	*C. dromedarius*	20	1 y	Healthy	Supporting HR/echocardiography: baseline	Echocardiography; clinical HR
Shekidef and Saleh (2011) [16]	Egypt	RCT	*C. dromedarius*	6	Adult	Healthy	Supporting HR: epidural ketamine/xylazine or medetomidine	Clinical HR; ECG detail NR
Almubarak (2013) [17]	KSA	Non-RCT	*C. dromedarius*	7	4.8 y	Healthy	Supporting HR: lidocaine (3 mg/kg IV)	Clinical HR; ECG detail NR
Masoudifard et al. (2020) [11]	NR	Non-RCT	*C. dromedarius*	20	6–8 mo	Healthy	Supporting HR/hemodynamics: medetomidine or xylazine	Pulsed-wave Doppler; HR
Nath et al. (2023) [18]	NR	RCT crossover	*C. dromedarius*	6	4–5 y	Healthy	Supporting HR: dexmedetomidine (2.5 or 4 µg/kg IV)	Clinical HR; ECG detail NR
Nath et al. (2024) [19]	NR	RCT crossover	*C. dromedarius*	6	4–5 y	Healthy	Supporting HR: dexmedetomidine/butorphanol/ketamine	Clinical HR; ECG detail NR
El-Sayed et al. (2025) [20]	Egypt	Cross-sectional	*C. dromedarius*	20	17.1 ± 1.5 y	Healthy	Supporting HR/echocardiography: baseline	Doppler echocardiography; clinical HR

Sex-reporting note: sex was incompletely reported, and no included study was designed to compare male and female camels. No sex-specific effect estimate was produced.

Supplementary Table S3. SYRCLE Risk-of-Bias Judgments

Study	D1	D2	D3	D4	D5	D6	D7	D8	D9	D10
Peshin (1980) [5]	?	+	?	?	?	?	?	+	+	?
Sharma (1983) [7]	-	+	-	?	?	?	?	+	+	?
Evans et al. (1994) [4]	?	+	?	?	?	?	?	+	+	?
Al-Mubarak (2008) [9]	-	?	-	?	?	?	?	?	+	-
Shekidef and Saleh (2011) [16]	+	+	?	?	?	?	+	+	+	+
Almubarak (2013) [17]	-	+	-	?	?	?	+	+	+	?
Samimi and Azari (2017) [6]	-	+	-	?	?	?	?	+	+	+
Masoudifard et al. (2020) [11]	?	+	?	?	?	?	?	+	+	+
Samimi and Sanjarinejad (2021) [8]	+	+	?	?	?	?	+	+	+	+
Nath et al. (2023) [18]	+	+	?	?	?	?	-	+	+	+
Nath et al. (2024) [19]	+	+	?	?	?	?	-	+	+	+

D1 = sequence generation; D2 = baseline characteristics; D3 = allocation concealment; D4 = random housing; D5 = blinding of caregivers/investigators; D6 = random outcome assessment; D7 = blinding of outcome assessor; D8 = incomplete outcome data; D9 = selective outcome reporting; D10 = other sources of bias. Low risk = green (+); high risk = red (-); unclear risk = yellow (?).
